# Dalteparin, a low-molecular-weight heparin, promotes angiogenesis mediated by heparin-binding VEGF-A *in vivo*

**DOI:** 10.1111/j.1600-0463.2010.02635.x

**Published:** 2010-12

**Authors:** KLAS NORRBY, ARVID NORDENHEM

**Affiliations:** 1 Department of Pathology, University of GothenburgGothenburg; 2 Biovitrum ABStockholm, Sweden

**Keywords:** Angiogenesis, low-molecular-weight heparin, dalteparin, epirubicin, VEGF-A, metronomic chemotherapy

## Abstract

Norrby K, Nordenhem A. Dalteparin, a low-molecular-weight heparin, promotes angiogenesis mediated by heparin-binding VEGF-A *in vivo*. APMIS 2010; 118: 949–57.

Tumors are angiogenesis dependent and vascular endothelial growth factor-A (VEGF-A), a heparin-binding protein, is a key angiogenic factor. As chemotherapy and co-treatment with anticoagulant low-molecular-weight heparin (LMWH) are common in cancer patients, we investigated whether angiogenesis *in vivo* mediated by VEGF-A is modulated by metronomic-type treatment with: (i) the LMWH dalteparin; (ii) low-dosage cytostatic epirubicin; or (iii) a combination of these two drugs. Using the quantitative rat mesentery angiogenesis assay, in which angiogenesis was induced by intraperitoneal injection of very low doses of VEGF, dalteparin sodium (Fragmin®) and epirubicin (Farmorubicin®) were administered separately or in combination by continuous subcutaneous infusion at a constant rate for 14 consecutive days. Dalteparin was administered at 27, 80, or 240 IU/kg/day, i.e., doses that reflect the clinical usage of this drug, while epirubicin was given at the well-tolerated dosage of 0.4 mg/kg/day. While dalteparin significantly stimulated angiogenesis in an inversely dose-dependent manner, epirubicin did not significantly affect angiogenesis. However, concurrent treatment with dalteparin and epirubicin significantly inhibited angiogenesis. The effect of dalteparin is the first demonstration of a proangiogenic effect of any LMWH *in vivo.* The fact that co-treatment with dalteparin and epirubicin significantly inhibited angiogenesis suggests a complex drug effect.

Tumor growth is angiogenesis dependent. Heparin-binding vascular endothelial growth factor-A (VEGF-A; VEGF) is a key angiogenic factor in hypoxia, wound healing, inflammation, and tumor development (1). VEGF also appears to be a mediator of angiogenic pathways involving other proangiogenic factors. The most potent endogenous pro- and anti-angiogenic proteins bind heparin and are dependent upon heparan sulfate for their biologic activities, while several enzymes and extracellular proteins bind heparin (2). Systemic heparin treatment may affect these proteins and enzymes, blood cells and other circulating cells, as well as vascular endothelial cells (ECs).

As tumor cells possess the capacity to interact with the hemostatic system, patients with cancer experience hypercoagulability with increased disseminated intravascular coagulation and thromboembolism, which requires anticoagulant therapy. In clinical trials, cancer patients undergoing surgery or suffering from acute thrombosis and receiving conventional chemotherapy in combination with s.c. administration of various low-molecular-weight heparins (LMWHs) display significantly improved survival times, as compared with patients who are treated either subcutaneously with unfractionated heparin (UFH) or orally with the anticoagulant warfarin, or those who do not receive any anticoagulant treatment (3–9). Extended survival is the most rigorous clinical benchmark for cancer drugs. The fact that LMWHs exert probably multiple anti-cancer activities that are basically unrelated to their anticoagulant activity attracts increasing interest as the basis for improved survival times in patients with cancer (2–6, 10). However, definitive conclusions regarding the true anti-neoplastic effects of LMWHs cannot be drawn from the data of clinical trials given the current methodologic limitations.

The marketed anticoagulant LMWHs represent a diverse group of depolymerized heparin preparations, with mean molecular weight (MW) ranging from 2 to 9 kDa, although they are usually in the approximately 6 kDa range (11). Dalteparin has a MW of 6 kDa. Most LMWHs are isolated from UFH (MW ∼15 kDa). Depending on the manufacturing processes used, the LMWHs show structural, biochemical, and pharmacologic differences, and their behaviors show dependencies on dosage and route of administration (12, 13). Regulatory agencies, such as the European Medicines Agency and the U.S. Food and Drug Administration, consider the LMWHs to be distinct pharmacologic agents and recommend that they should not be regarded as being interchangeable (14, 15). However, the pharmacologic differences between these agents do not produce clinically important differences in the outcomes of anti-coagulation therapy in patients with venous thromboembolism (14).

Metronomic chemotherapy, which is characterized by continuous or frequent treatment with cytotoxic agents at low and well-tolerated dosages, has for many drugs been shown in pre-clinical studies to exert potent anti-angiogenic effects and improved anti-tumor effects without important toxic side effects, as compared with conventional chemotherapy given at high doses at intervals of typically 2–4 weeks (16, 17). Somewhat similar beneficial results are reported concerning metronomic chemotherapy in patients with common types of cancer (18–21). Continuous infusion, as used in the present study, can be viewed as an extended form of metronomic treatment (22). The primary target of metronomic chemotherapy is the angiogenically activated, proliferating normal EC in the tumor vasculature. The activated EC is genomically stable, and therefore, is endowed with a considerably lower risk of mutation and subsequent development of drug resistance than genomically unstable neoplastic cells. In addition to exerting a direct anti-proliferative effect on angiogenically activated ECs, some chemotherapeutics induce the expression of the potent endogenous anti-angiogenic factor thrombospondin in ECs cells *in vitro* (23) and tumors *in vivo* (24).

The aim of the present study was to elucidate whether metronomic-like treatment, i.e., continuous s.c. infusion, with the dalteparin alone; the chemotherapeutic agent epirubicin alone at a low, virtually non-toxic dose; or these two drugs in combination modulates VEGF-mediated angiogenesis *in vivo*.

## Materials and methods

### Animals

Adult male Sprague–Dawley rats (B & K Universal, Sollentuna, Sweden) were acclimatized to a standardized environment for at least 7 days, fed *ad libitum* and randomly allocated to weight-matched groups with two animals housed per cage (25). At the start of the experiments, when the animals were 6–7 weeks old, the mean body weights in different experiments ranged from 218 to 223 g. Body weight was monitored daily. The controls increased in weight by approximately 55 g per week. Given the prompt physiologic growth, drug-related weight-gain retardation is a sensitive surrogate evaluation of toxicity, which also includes systemic well-being, anorexia and failure to thrive. Gauging body weight gain during chemotherapy is important, as low toxicity, which allows long-term continuous or frequent treatment, is inherent to metronomic scheduling. Moreover, toxic effects diminish analysis sensitivity. The local Animal Ethics Committee approved this study. The ethical guidelines followed meet the standards set by the UKCCCR (26).

### Angiogenesis induction and a note on the mesentery assay

Rat rVEGF_164_ (564-RV/CF; R&D Systems Europe, Ltd., Oxon, UK), which is the predominant VEGF-A isoform in rats, was diluted to 96 pmol/mL in endotoxin-free saline used for infusion into patients, frozen and thawed, and a volume of 5 mL was injected i.p. into the rats (27). This treatment, given twice daily for 4.5 days, i.e., from Monday morning (Day 0) to Friday morning (Day 4), induces a vigorous angiogenic response in the mesenteric test tissues, peaking around Day 21 (27). The VEGF does not affect body weight gain. It was within this time frame of microvessel network proliferation that the s.c. treatments with dalteparin and epirubicin were given.

Similar to most normal adult tissues, the test tissue used, i.e., the membranous, small-gut mesentery in rats is natively vascularized (albeit sparsely) and lacks significant physiologic angiogenesis due to equilibrium between pro- and anti-angiogenic influences (28, 29). The test tissue is untouched mechanically until the experiment is concluded. The inflammatory stimulus of the test tissue is minimal, if any, ensuring a high level of sensitivity, because inflammation induces angiogenesis (28). This model compares well with other *in vivo* angiogenesis models, as discussed elsewhere, and allows true quantification of unbiased variables (28, 29). Importantly, the rat mesenteric assay replicates the clinical situation, as the test drugs are administered systemically and the responses observed reflect the net effect of all the metabolic, cellular, and molecular alterations induced by the treatment.

### Continuous subcutaneous infusion of dalteparin and epirubicin for 14 days

#### Filling and implanting of osmotic minipumps–

On Day −2, i.e., 2 days before the start of the angiogenic i.p. VEGF treatment, osmotic minipumps (Model 2002 for dalteparin and Model 2ML2 for epirubicin, with constant pumping rates of 0.5 and 5.0 μL/h for 14–15 days, respectively; Alzet® Osmotic Pumps, Mountain View, CA, USA) were filled under sterile conditions with the test solution or its vehicle. One day later (Day −1), after being stored in sterile 0.9% (w/v) NaCl overnight at 37 °C, the pumps were surgically implanted s.c. on the backs of rats that had been anesthetized with inhaled isoflurane (Forene®, Abbott, Abbott Park, IL, USA). The skin incision, made for pump implantation, was immediately sutured post-implantation. As only minute volumes were infused at a low rate, it is highly improbable that pH in the mesentery microvessels was affected to any measurable degree by the difference in vehicle pH for dalteparin and epirubicin. As the animals substantially gained weight physiologically during the experimental period (Table 1), the actual dose per kg body weight was somewhat above the average dosage at the beginning of the infusion period and somewhat below the average dosage at the end of the infusion period.

**Table 1:** Effect on physiologic body weight gain of continuous subcutaneous epirubicin infusion for 14 consecutive days

Body weight, g				
Day	Vehicle control	Epirubicin, mg/kg/week		
		1.5	3.0	6.0
		Percentage of vehicle control		
0	225.0 ± 2.6	222.3 ± 2.5	225.0 ± 2.2	218.5 ± 3.4
	100	99	100	97
6	275.0 ± 4.2	271.7 ± 3.4	268.2 ± 2.0	255.3 ± 2.3
	100	99	98	93
9	297.3 ± 5.0	287.2 ± 3.6	289.0 ± 3.2	272.0 ± 2.7
	100	97	97	91
14	334.2 ± 5.6	318.3 ± 4.9	314.8 ± 4.4	291.7 ± 2.9
	100	95	94	87

Each treatment group comprised six animals. The vehicle was 0.9% NaCl. The vehicle control was set at 100%. Data shown are mean ± SEM.

#### Dalteparin monotherapy–

A dose–effect study of continuously infused dalteparin at 27, 80, and 240 IU/kg/day, which is within the dose range used in patients, on VEGF-mediated angiogenesis was performed (Table 2). The vehicle saline (pH 7) is used for infusion into patients. Dalteparin at 80 IU/kg/day (Fragmin®, Pfizer, New York, NY, USA; mean molecular weight range 5.6–6.4 kDa, with MW of 6.0 kDa; degree of sulfation, 2.0–2.5 per disaccharide unit) was infused (Table 2). Vehicle controls were included.

**Table 2:** Effect of 14 days of subcutaneous continuous infusion of dalteparin alone, epirubicin alone, and a combination of the two drugs on VEGF-mediated angiogenesis and body weight (BW) at sacrifice

	Vehicle/D + Vehicle/E^2^	Dalteparin^1^ + Vehicle/D^2^	Epirubicin^1^ + Vehicle/E	Dalteparin + Epirubicin
	Group C I	Group C II	Group T I	Group T II
BW, g	323.30 ± 6.20 (100)	322.00 ± 4.76 (100)	299.60 ± 4.94 (93)	301.40 ± 5.59 (93)
VA^3^	14.55 ± 2.36 (100)	18.47 ± 2.89 (127)	14.68 ± 2.00 (101)	13.25 ± 2.20 (91)
MVL	1.12 ± 0.08 (100)	1.15 ± 0.07 (103)	0.98 ± 0.08 (88)	0.88 ± 0.06* (79)
TMVL	16.28 ± 2.64 (100)	21.28 ± 3.33 (131)	14.39 ± 1.96 (88)	11.64 ± 1.94** (71)

Values within parenthesis are % of value for CI.

1 Dalteparin at 80 IU/kg/day; epirubicin at 3.0 mg/kg/week.

2 Vehicle/D, 0.9% NaCl; Vehicle/E, 0.9% NaCl. Each treatment group comprised 10 animals, with the exception of group T II, which comprised nine animals (one rat was removed due to signs of sickness). There are no statistically significant differences with respect to any variable between groups T I and T II or between groups C I and C II. Data shown are mean ± SEM.

3 VA, vascularized area, a measure of microvessel spatial extension; MVL, microvascular length, a composite measurement of microvessel density; TMVL, total microvascular length (VA × MVL).

* p ≤ 0.02 compared with C II, p ≤ 0.01 compared with [C I + C II];

** p ≤ 0.04 compared with C II, p ≤ 0.05 compared with [C I + C II].

#### Epirubicin monotherapy–

In an initial dose-finding experiment, epirubicin, which is of the anthracycline class of intercalating topoisomerase-targeting agents (30), was infused at doses of 1.5, 3.0, and 6.0 mg/kg/week. The drug was diluted in 0.9% (w/v) NaCl (pH 4.5), used for infusion into patients. Vehicle controls were infused in a similar fashion. The intention was to find a well-tolerated dose that would only marginally diminish the weight gain, as compared with the rapidly growing vehicle controls (Table 1). In the subsequent angiogenesis experiments, epirubicin at 3.0 mg/kg/week was used. It has been claimed that the dosage per body surface area (mg/m^2^) may be useful in comparing drug toxicities between species (i.e., between laboratory animals and humans). For a rat that weighs 250 g, the dosage in mg/kg multiplied by 7 yields the approximate dosage in mg/m^2^. Thus, the dose used in the angiogenesis experiment corresponds to ∼21 mg/m^2^ per week in a human.

#### Dalteparin and epirubicin co-treatment–

The animals were infused with epirubicin at 3.0 mg/kg/week and dalteparin at 80 IU/kg/day or vehicle, using two minipumps per animal (Table 3).

**Table 3:** Effects on VEGF-mediated angiogenesis of continuously infused dalteparin administered subcutaneously at different doses for 14 consecutive days

Dalteparin IU/kg/day	Angiogenesis variables		
	VA^1^	MVL	TMVL
Vehicle control (n = 12)	11.72 ± 2.66 (100)	0.901 ± 0.100 (100)	10.56 ± 2.40 (100)
27 (n = 12)	16.07 ± 2.67 (137)	1.126 ± 0.072** (125)	18.09 ± 3.00** (171)
80 (n = 11)	16.89 ± 3.82 (144)	1.064 ± 0.107 (118)	17.26 ± 3.90* (163)
240 (n = 9)	13.51 ± 1.93 (115)	0.872 ± 0.076*** (97)	11.78 ± 1.68 (112)

Values within parenthesis are percent of control.

1 VA, a measurement of microvessel spatial extension; MVL, a composite measurement of microvessel density; TMVL = (VA × MVL).

* p ≤ 0.10,

** p ≤ 0.05 compared with vehicle (0.9% NaCl) control;

*** p ≤ 0.05 compared with the low dose of 27 IU/kg/day. Data shown are mean ± SEM. n = number of animals.

On Day 10 of the 14-day treatment, one animal in the 80 IU/kg/day group and three animals in the 240 IU/kg/day group developed s.c. blood-streaked liquid accumulation at the site of the osmotic infusion pump and were therefore sacrificed. These animals are excluded from the analysis.

### Angiogenesis quantification

After 14 days of treatment, four membranous, virtually transparent (window-like) samples from the most distal part of the small-gut mesentery, immediately proximal to the ileocecal valve, were examined microscopically after being spread intact on objective slides and stained immunohistochemically (27). Normally, this tissue is only 5- to 10-μm thick in its avascular parts, making the whole microvessel network virtually two-dimensional, which is the basis for the unique ability to quantify microvessel variables in the intact tissue. The surrounding fatty tissue distinctly delineated each window. The entire vasculature of each of the four mesenteric windows per animal was visualized using a primary monoclonal antibody against rat endothelium, MRC OX-43 (31), which labels the vascular endothelium in all tissues of the rat, except that of the brain capillaries. This procedure allows the straightforward identification of even the smallest microvessels.

For the analysis of unbiased microvessel variables, microscopic morphometry and computerized image analysis were employed in a blinded fashion. Objective variables are a prerequisite for strict dose–response analysis. First, the total area of each mesenteric window was measured. The following variables were then measured in each window (27): the percentage vascularized area (VA), which is a measurement of the spatial extension of the network; and microvascular length (MVL), which is a composite measurement of microvessel density. The total microvascular length (TMVL) was calculated as TMVL = VA multiplied by the mean MVL/treatment group.

### Statistical analysis

The non-parametric Mann–Whitney *U*-test for unpaired (two-tailed) observations was used. A mean of four windows per animal was used as independent data for each variable in the mesenteric window. The criterion for statistical significance was p ≤ 0.05.

## Results

### Effect on physiologic body weight gain of dalteparin and epirubicin treatment

Continuous infusion of dalteparin at 27, 80, and 240 IU/kg/day for 14 days did not affect body weight gain compared with vehicle controls (data not shown). The effects of the continuous infusion of epirubicin at 1.5, 3.0, and 6.0 mg/kg/week are shown in Table 1. Based on previous experience (27, 28), we concluded that epirubicin at 3.0 mg/kg/week, which reduced body weight by only 6% at sacrifice compared with the rapidly growing vehicle-treated controls, was the appropriate dosage.

### Effects on VEGF-mediated angiogenesis of continuous infusion of dalteparin alone, epirubicin alone, and co-treatment of dalteparin and epirubicin

Treatment with dalteparin (80 IU/kg/day) + vehicle (group C II), using two s.c. pumps per animal, increased overall angiogenesis TMVL by 31%, as compared with the controls that were treated with vehicle + vehicle (group C I) using two s.c. pumps (Table 2). However, this difference was statistically non-significant. Treatment with epirubicin + vehicle (group T I) reduced the TMVL by 12%, as compared with the vehicle + vehicle control (group C I), although this difference was also non-significant. Co-treatment with dalteparin + epirubicin (group T II) reduced the MVL by 24% (p ≤ 0.02) and TMVL by 45% (p ≤ 0.04), as compared with the controls that received dalteparin + vehicle (group C II). Furthermore, treatment with dalteparin + epirubicin (group T II) significantly reduced both the MVL and TMVL, as compared with the extended control group (vehicle + vehicle and dalteparin + vehicle, i.e*.,* groups C I and C II), as shown in Table 2.

As the infusion of dalteparin (80 IU/kg/day) + vehicle for 14 days increased the TMVL by 31%, compared with the vehicle + vehicle controls (Table 2), we performed a dose–effect study, in which we infused dalteparin continuously for 14 days at 27, 80, and 240 IU/kg/day (Table 3). The lowest dose, 27 IU/kg/day, increased the VA by 37%, MVL by 25%, and TMVL by 71% (p ≤ 0.05), compared with the corresponding values for the vehicle control. The highest dosage, 240 IU/kg/day, had only a marginal effect on angiogenesis, while 80 IU/kg/day exerted intermediate effects on the VA (+44%), MVL (+18%), and TMVL (+63%). It is noteworthy that the effect of dalteparin on MVL was significantly dose dependent (Table 3).

## Discussion

The present study demonstrating that dalteparin (MW, 6 kDa) stimulates VEGF-mediated angiogenesis is to the best of our knowledge the first report of a proangiogenic effect of any LMWH *in vivo* (2). By contrast, a 5-kDa fraction of the LMWH tinzaparin (Innohep®, LEO Pharma, Copenhagen, Denmark) has been shown to inhibit VEGF-induced angiogenesis *in vivo* using the same experimental system (2, 25). As dalteparin and tinzaparin display similar degrees of sulfation and the difference in MW (6 kDa vs 5 kDa) is small, it appears that the particular type of LMWH rather than the mean molecular weight is decisive for the outcome in the angiogenesis model used. This is important because the anti-angiogenic effect of tinzaparin is dependent on molecular size (25). While dalteparin is produced by nitrous acid depolymerization of porcine gut mucosal UFH, which results in the formation of anhydromannose (a five-member ring) in the molecule, tinzaparin is produced by controlled enzymatic depolymerization by heparinase digestion of porcine gut mucosal UFH, which adds a double bond to the end group of the molecule (12, 15, 32). Interestingly, heparin fragments in the 4.8–5.4 kDa range, produced from porcine gut mucosa UFH after being dissolved in buffer and exposed to chromatography on a sephadex column, inhibit the binding of ^125^I-VEGF to the VEGF receptors on cultured ECs (33).

As tumor growth is angiogenesis dependent, it is of interest to assess the anti-angiogenic effects of candidate anti-tumor drugs. However, there are no ways to accurately assess the anti-angiogenic effect *per se* of drugs on tumors because inhibition of angiogenesis limits tumor growth and vice versa (28, 34, 35). The true anti-angiogenic effect of any treatment must therefore be assessed in a surrogate non-tumor tissue. As VEGF is a key proangiogenic factor in most human and experimental tumors (1), VEGF-mediated angiogenesis would be an appropriate surrogate model for tumor angiogenesis, especially because the ECs in tumor angiogenesis use the same signaling pathways as the ECs involved in non-tumor angiogenesis (36).

However, the present assay does not take into account all the aspects of tumor-induced angiogenesis, such as the presence of multiple proangiogenic factors that usually operate in advanced cancers (37) or the chaotic microvessel patterns and perfusion of tumor vasculature observed predominantly in the central parts of tumors. Nevertheless, the present assay for VEGF-mediated angiogenesis has demonstrated a close correlation between the anti-angiogenic effects in the tumor-free mesentery on one hand and the anti-tumor and indirectly assessed anti-angiogenic effects in the tumors on the other hand after continuous paclitaxel infusion in a rat model of syngeneic prostate cancer (38). Additional corroboration that inhibition of VEGF-induced angiogenesis in the tumor-free mesentery may reflect significant events leading to inhibition of tumor growth comes from the fact that systemic treatment with bovine iron-unsaturated lactoferrin suppresses VEGF-induced angiogenesis in the rat mesentery model (39), as well as cancer cell-induced angiogenesis in a mouse dorsal air sac assay (40). Compared with other non-tumor angiogenesis assays, the present assay stands out as apparently superior in terms of biologic relevance and ability to objectively quantify pertinent angiogenesis variables (28, 29).

The fact that the continuous treatment with (dalteparin + epirubicin) inhibited angiogenesis significantly as compared with (dalteparin + vehicle) or (epirubicin + vehicle) treatment is indeed interesting in view of the improved survival time of cancer patients who receive co-treatment of dalteparin and other LMWHs with conventional chemotherapy (3–8, 10). As is true for heparins in general, dalteparin is able to bind, release, and influence the actions of heparin-binding proteins, such as proangiogenic growth factors (e.g., VEGF and basic fibroblast growth factor), anti-angiogenic factors (e.g., thrombospondin), enzymes (e.g., heparanase), and extracellular matrix proteins that may significantly influence angiogenesis (2, 12, 41, 42). Moreover, heparins influence a number of additional angiogenesis-modulating factors, such as P- and L-selectins, integrins, platelets and all other circulating cells, circulating microparticles (derived from ECs and leukocytes), antioxidant function, and release tissue factor pathway inhibitor (TFPI-1). TFPI-1, which is mainly synthesized in and localized to ECs, is the natural inhibitor of tissue factor, which is a critical promoter of angiogenesis. It is noteworthy that different LMWHs exhibit major differences in their abilities to release TFPI from the vascular lining (43), the highest release occurring following treatment with tinzaparin (10, 43). Not surprisingly, TFPI-1 released by 6–8 kDa tinzaparin fractions suppresses angiogenesis in the embryonic chick chorioallantoic membrane assay (44).

As recently demonstrated using the present experimental system, low concentrations of exogenous antioxidants play a decisive role in the angiogenesis-modulating effects of certain cytotoxic agents that are infused continuously at low doses (31, 45). Thus, fluorouracil, cisplatin, and paclitaxel significantly inhibit VEGF-mediated angiogenesis when the vehicle displays antioxidant activity or in the case of co-treatment with a potent antioxidant, whereas these cytotoxics show no anti-angiogenic effects when the vehicle lacks an antioxidant. By contrast, continuous low-dosage infusion of doxorubicin, which is an epimer (one of two molecules that differ only in the spatial arrangement around a single carbon atom) of epirubicin, does not affect VEGF-mediated angiogenesis even when combined with an antioxidant (31). Therefore, the anti-angiogenic effects by the concurrent treatment with dalteparin and epirubicin (Table 2) are probably not dependent on antioxidant activity of dalteparin. Some additional, as yet unknown, feature of dalteparin appears to play an important role in the anti-angiogenic action. The fact that (dalteparin + epirubicin) significantly inhibited angiogenesis, while the individual drugs at the doses given did not influence angiogenesis, is in line with results obtained for mice bearing the human U87 glioblastoma xenograft: in these mice, tumor growth was unaffected by daily s.c. injections of dalteparin alone or only moderately affected by SU5416 alone, whereas the co-treatment of dalteparin and SU5416 significantly decreased the level of VEGF in the tumors and strongly inhibited tumor growth (46). SU5416 is a small molecule inhibitor of tyrosine kinase receptors, including the VEGF receptors.

As it is not known which of the marketed LMWHs exerts the most potent anti-neoplastic effect in cancer patients receiving chemotherapy, be it conventional or metronomic, bona fide prospective clinical trials comparing directly the effects of two or more LMWHs on survival time and disease progression are warranted.

The proangiogenic effect of dalteparin in the present report should be beneficial for the treatment of non-cancer patients with poor peripheral circulation or thromboembolism causing tissue hypoxia, which induces compensatory VEGF-mediated angiogenesis. The present findings actually appear to fit the available clinical data in this respect. It is of note that daily s.c. injections of dalteparin for up to 6 months (5,000 IU, corresponding to ∼60–70 IU/kg/day) significantly improve the outcomes for chronic foot ulcers in diabetic patients with peripheral arterial occlusive disease (47). Furthermore, it has been hypothesized that dalteparin, which appears to improve the capillary circulation in the foot ulcer margin, could have a proangiogenic effect in these patients (48). Moreover, in patients with stable coronary artery disease, exercise in combination with dalteparin reduces myocardial ischemia, possibly as a result of enhanced collateral function (49).
